# Gene-methylation epistatic analyses via the W-test identifies enriched signals of neuronal genes in patients undergoing lipid-control treatment

**DOI:** 10.1186/s12919-018-0143-8

**Published:** 2018-09-17

**Authors:** Rui Sun, Haoyi Weng, Ruoting Men, Xiaoxuan Xia, Ka Chun Chong, William K. K. Wu, Benny Chung-Ying Zee, Maggie Haitian Wang

**Affiliations:** 1 Division of Biostatistics, Centre for Clinical Research and Biostatistics, JC School of Public Health and Primary Care, the Chinese University of Hong Kong, Shatin, N.T., Hong Kong, Hong Kong, Special Administrative Region of China; 2 The Chinese University of Hong Kong Shenzhen Research Institute, Shenzhen, China; 3 Department of Anaesthesia and Intensive Care, the Chinese University of Hong Kong, Shatin, N.T., Hong Kong, Hong Kong, Special Administrative Region of China

## Abstract

An increasing number of studies are focused on the epigenetic regulation of DNA to affect gene expression without modifications to the DNA sequence. Methylation plays an important role in shaping disease traits; however, previous studies were mainly experiment, based, resulting in few reports that measured gene–methylation interaction effects via statistical means. In this study, we applied the data set adaptive W-test to measure gene–methylation interactions. Performance was evaluated by the ability to detect a given set of causal markers in the data set obtained from the GAW20. Results from simulation data analyses showed that the W-test was able to detect most markers. The method was also applied to chromosome 11 of the experimental data set and identified clusters of genes with neuronal and retinal functions, including *MPPED2I, GUCY2E*, *NAV2*, and *ZBTB16*. Genes from the *TRIM* family were also identified; these genes are potentially related to the regulation of triglyceride levels. Our results suggest that the W-test could be an efficient and effective method to detect gene–methylation interactions. Furthermore, the identified genes suggest an interesting relationship between lipid levels and the etiology of neurological disorders.

## Background

Genetic variants, such as single-nucleotide polymorphisms (SNPs), have been found to influence risk for human diseases. Recent studies show that epigenetics affect SNPs in genes and subsequently influence the gene function and disease trait [1]. Epigenetic mechanisms consist of DNA methylation, histone modifications, and noncoding RNAs, all of which represent the patterns of chemical and structural modifications to DNA [2]. There are an increasing number of laboratory experiments that provide evidence of DNA methylation and gene expression regulation [3–5]. Only a few studies, however, have evaluated the genome–epigenome interactions through statistical means, which may potentially provide novel findings for the joint effects of SNPs and cytosine-phosphate-guanine (CpG) sites [6–9]. The search for SNP-CpG epistasis is usually conducted through multistage or integrated analyses, where the genome and methylation data are first analyzed separately and the results then combined [10, 11]. Some studies apply existing interaction-effect methods, such as regressions, to perform the joint analysis of methylation and genome data. The advantages of the W-test method are data set adaptive probability distributions and robustness for complicated genetic architectures, such as moderate data sparsity and population stratifications [12]. By applying the W-test to gene–methylation data directly, epistasis can be measured without a preselection of biomarkers, while also relying less on significant main effects for detecting important CpG–SNP interactions. The GAW20 provided an opportunity to study methylation and genome-wide association study (GWAS) data from participants who have undergone lipid control treatment. The W-test was applied in the detection of gene–methylation interactions, resulting in interesting findings with biological implications.

## Methods

### GAW20 experimental and simulated data sets

GAW20 provided the study data. The study participants were patients with diabetes who had undergone lipid-control treatments with the drug fenofibrate and were recruited from the Genetics of Lipid Lowering Drugs and Diet Network (GOLDN) clinical trial project. The analyzed data sets consisted of a simulated and experimental data sets. The triglyceride (TG) levels were collected at 4 clinical visits, with 2 measurements before treatment and 2 measurements after treatment. Age, sex, smoking status, and location were recorded. Genome-wide association data were sequenced with the Affymetrix Genome-wide Human SNP array 6.0, and DNA methylation profiling was performed with the Illumina Infinium Human Methylation 450 K Bead Chip Array, using the buffy coat harvest from blood samples collected at the second and fourth visits. In the simulated data, the phenotype of the simulated data set was generated using experimental genetic data under a hypothetical model [13]. The TG levels were generated from 5 SNPs with major effects and 5 CpG sites in physical proximity. A set of 5 SNP-CpG pairs with relatively high heritability but not related to TG levels was given as noise for testing the statistical methods. The simulated data contained 680 subjects after excluding individuals with missing phenotypes. For simulated data, the 84th replicate was used as suggested by GAW20. In the experimental data set, a total of 523 participants had complete genomic and clinical measurements. Participants with missing values were removed during the quality-control process, resulting in a remaining sample size of 476. The method was applied to chromosome 11 of the experimental data.

### Defining drug response

The TG levels can be used as a measure of drug response. Because common clinical standards regard a 30% decrease in TG levels as an effective control of lipids [14], we adopted the same criteria in this study. First, the average pretreatment TG levels (TG_pre) were calculated by averaging the measurements from the first and second visits. The average posttreatment TG levels (TG_post) were calculated by averaging the measurements from the third and fourth visits. Next, a percentage of change was calculated as: ΔTG% = (TG_pre–TG_post)/TG_pre. If the percentage of change was greater than 30%, then the drug treatment was defined as effective; if less than 30%, treatment was defined as ineffective. The effectiveness of the drug response was the outcome variable for both the simulated and experimental data.

### The epistasis measure: The W-test

The W-test measures the probability distributional differences for a set of biomarkers between the 2 groups of participants such as the 2 drug-response groups [12]. Under an additive genetic model, a SNP variable can be coded into 3 levels with the counts of the minor alleles. The quantitative CpG variable can be divided into high and low methylation levels by two-mean clustering. A SNP-CpG pair can form a genetic combination of 6 categories. The empirical distributions are compared through a sum of the square of the log odds ratio by:

1 $$ W=h\sum \limits_{i=1}^k{\left[\log \frac{{\widehat{p}}_{1i}/\left(1-{\widehat{p}}_{1i}\right)}{{\widehat{p}}_{0i}/\left(1-{\widehat{p}}_{0i}\right)}/{SE}_i\right]}^2\sim {\chi}_f^2 $$

where ${\widehat{p}}_{1i}$ and ${\widehat{p}}_{0i}$ are the proportion of cases and controls in the *i*^*th*^ category out of total cases or controls, respectively. *SE*_*i*_ is the standard error of the log of odds ratios. The test statistics follows a chi-squared distribution with *f* degrees of freedom. Two parameters, *h* and *f*, are estimated using large-sample approximation by drawing smaller bootstrap samples under a null hypothesis. Consequently, the testing distribution is robust for complicated genetic architectures, as it adaptively adjusts to the data structure of the working data [12]. For detecting the *cis*-regulation patterns in the gene-methylation data, the SNPs and CpG sites located within a 10-kb genome distance on chromosome 11 were evaluated exhaustively [1].

Two types of logistic-regression models were applied as accompanying benchmarks to the W-test. The first logistic-regression model, LR-m1, considered the CpG site as a binary variable like the W-test, and the second logistic-regression model, LR-m2, included the CpG sites as a continuous variable using the original methylation values. Both logistic-regression models incorporated the main and interaction effects of SNP and CpG sites. In short, we denote:

- LR-m1: *Y* = SNP + CpG + SNP × CpG, where CpG is a binary variable;
- LR-m2*: Y* = SNP + CpG + SNP × CpG, where CpG is a continuous variable.

The type I error rate is an average false-positive proportion using a permuted phenotype on a pair of gene–methylation markers in 2000 replicates. A total of 140,501 epistatic pairs were tested, and a Bonferroni correction resulted in a significance level of 3.56E-7 at a family-wise error rate of 5%.

## Results

### Performance of the W-test with simulated data

In the simulated data set, the W-test, LR-m1, and LR-m2 were applied to the given causal and noise pairs. Table 1 displays the *p* values obtained from alternative methods. Generally, the W-test gave smaller *p* values than LR-m1 in most answer pairs, and also had comparable *p* values to LR-m2. The top 3 answer pairs were all identified to be significant by the 3 methods. The W-test also found the fourth answer pair (cg00045910, rs10828412) with a *p* value = 0.0475, which was slightly smaller than the *p* values from the LR-m1 (*p* value = 0.0532) and LR-m2 (*p* value = 0.0597). The results suggested that the W-test could be sensitive to small signals with lower heritability. In terms of the performance for the noise pairs, all methods yielded noise *p* values greater than 0.05. The Type I error rate of the W-test was 2.95%, less than the family-wise error rate of 5%. Meanwhile, the Type I error rates of LR-m1 was 5.40% and of LR-m2 was 5.43%. The results showed that the W-test was able to distinguish between signal and noise in the simulated data set.

**Table 1:** *p* Values of 5 answers and 5 noises by the W-test and the logistic regression models LR-m1 and LR-m2 in simulated data

	No	Marker information				*p* Value		
		CpG	SNP	Heritability	Chr	W-test	LR-m1	LR-m2
Answer	1	cg00000363	rs9661059	0.125	1	4.93E − 5	1.88E − 4	2.37E − 5
	2	cg10480950	rs736004	0.075	6	6.61E − 4	2.17E − 3	3.72E − 4
	3	cg18772399	rs1012116	0.1	8	7.67E − 4	2.04E − 4	8.24E − 4
	4	cg00045910	rs10828412	0.025	10	4.75E − 2	5.32E − 2	5.97E − 2
	5	cg01242676	rs4399565	0.05	17	3.76E − 1	6.33E − 1	4.95E − 1
Noise	6	cg00703276	rs2953763	–	3	5.11E − 1	1.84E − 1	1.32E − 1
	7	cg01971676	rs6960763	–	7	6.30E − 1	6.72E − 1	4.19E − 1
	8	cg11736230	rs2494731	–	14	1.61E − 1	2.06E − 1	1.10E − 1
	9	cg00001261	rs4786421	–	16	4.18E − 1	1.46E − 1	5.56E − 1
	10	cg12598270	rs323312	–	18	7.33E − 1	8.03E − 1	4.19E − 1

### Computing time

Computing time was calculated on a laptop computer with a 1.6 GHz chipset and 4 GB of random access memory using 2000 replicates on 1 pair of markers. The W-test was 4 times faster than logistic regression on a general laptop (2.28 s by the W-test, 10.12 s by the LR-m1, and 9.37 s by the LR-m2).

### Identification of gene–methylation interaction in experimental data

**Fig. 1:**
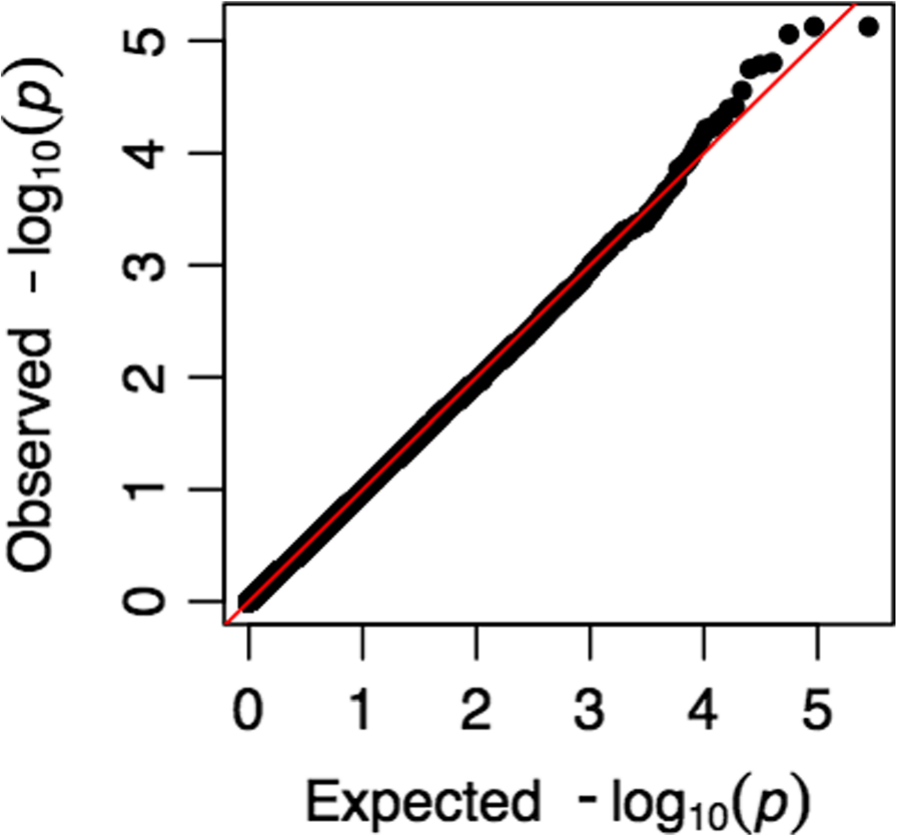
Q-Q plot of gene–methylation interaction using experimental data

The W-test was applied to test the gene–methylation interactions for GAW20 experimental data on chromosome 11. No significant interaction pair passed the Bonferroni correction significance level of 3.56E-07 (Table 2). We checked the functions of the top 15 identified epistatic pairs and found interesting biological implications. The top 3 SNP-CpG pairs all resided in the gene *MPPED2* (11p14.1; *p* value = 8E-06), which encoded the protein metallophosphoesterase and was reported to be related to neuronal function [15]. Previous GWAS studies and biomedical experiments reported that *MPPED2* was associated with chronic kidney disease, and knockdown of this gene in zebrafish embryos suggested a role for it in renal function [16]. *GUCY2E* was ranked fourth and has been reported to function in the central nervous system and retinal [17, 18]. *NAV2* (ranked 6th; *p* value = 1.78E-05) is a neuron navigator that induces neurite outgrowth for all-*trans* retinoic acid, and plays an essential role in the development of the cranial nerve and the regulation of blood pressure in humans [19]. *ZBTB16* at 11q23.2 (ranked 15th; *p* value = 7.04E-05) also has been reported as an inhibitor of neurite outgrowth in the adult central nervous system [20]. Other genes in the top 15 identified pairs include *TRIM5*, *TRIM6-TRIM34*, and *TRIM3* (smallest *p* value = 5.22E-05), which were highly correlated with TG levels in mice [21]. The quantile–quantile (Q-Q) plot of the gene–methylation tests showed no inflation in spurious relations for the experimental data (Fig. 1).

**Table 2:** Top 15 gene–methylation pairs identified by the W-test in experimental data^a^

	SNP	CpG	Distance (kb)	Gene	MAF	*p* Value
1	rs12288568	cg13342435	1.27	*MPPED2*	0.003	7.49E − 06
2	rs11031153	cg13342435	3.86	*MPPED2*	0.003	7.49E − 06
3	rs16921036	cg13342435	1.35	*MPPED2*	0.001	8.68E − 06
4	rs11237066	cg13340272	4.52	*GUCY2E*	0.120	1.57E − 05
5	rs7119411	cg17432267	3.75	*C11orf63*	0.430	1.65E − 05
6	rs11025246	cg08550026	8.63	*NAV2*	0.395	1.78E − 05
7	rs4347345	cg16454587	2.50	*–*	0.016	2.78E − 05
8	rs16927166	cg04054921	5.60	*TNNT3*	0.007	3.94E − 05
9	rs2165313	cg11007153	2.43	*B3GAT1*	0.237	4.06E − 05
10	rs11025246	cg04916810	9.60	*NAV2*	0.395	4.86E − 05
11	rs3740996	cg23217386	4.60	*TRIM5;TRIM6-TRIM34;TRIM3*	0.131	5.22E − 05
12	rs16921012	cg13342435	7.99	*MPPED2*	0.001	5.86E − 05
13	rs10895360	cg03879971	5.78	*LOC100128088*	0.024	6.04E − 05
14	rs900865	cg23454003	0.87	*–*	0.464	6.17E − 05
15	rs1455650	cg25744613	8.27	*ZBTB16*	0.155	7.04E − 05

^a^Bonferroni corrected significance threshold: 3.56E − 7

## Discussion and conclusions

There has been increasing evidence for the contribution of epigenetics in regulating gene expressions implicated in diseases. Previous studies were mainly focused on experimentally studying gene–methylation interactions. In this study, we demonstrated that the W-test can be used as an effective method to identify the epistatic interactions between SNPs and CpG sites in the GAW20 simulated and experimental data sets. One common obstacle in the analysis of epistasis in the genome and epigenome is the large number of pairwise tests, the volume of which is determined by the size of the *cis*-regulatory region. Existing methods solve the challenge by using stage-wise and integrated analyses, in which the SNPs are separately selected and then the epistatic interactions with CpG sites are jointly evaluated in regression-based approaches [10, 11]. The stage-wise analysis may potentially miss the markers that have weak main effects but strong epistasis effects. Previous studies also made a linear assumption about the relationship between the epistatic pairs and a transformed form of the response variable, while having the advantages of covariate and population structure control. Some nonparametric methods, such as the Mann-Whitney U-test, have been applied for the analysis of methylation data [22]. However, these nonparametric tests cannot handle the potential complicated genetic architectures such as sparse data or population stratification. The W-test has the advantage of being model-free and does not assume any form of interaction effect. It also follows a chi-squared distribution in which the degrees of freedom is estimated from the working data by bootstrapped sampling. In this way, the W-test is able to correct potential bias of the probability distribution caused by complicated data structures. This method is very efficient such that it can be applied directly on SNP-CpG evaluations without prior filtering with the main effect.

Application of this method on the experimental data from patients who had undergone treatment for managing TG levels via fenofibrate identified genes that played roles in renal function, the central nervous system, and retinal functions. The enriched signals found in neuronal-related genes suggest that the blood lipid levels could be related to the neurological dysfunction in the brain, which is the most cholesterol-rich organ in the body. By performing an epistatic evaluation between SNPs and CpG sites, we identified *MPPED2*, *GUCY2E*, *NAV2*, and *ZBTB16* as associated with hyperlipidemia. Among these 4 genes, *MPPED2* was the most significant; it plays a role in neural development, and genetic variations in this gene are reported to be related to migraines, a common disease of the neural system disease [23]. Furthermore, mutations of *CUCY2E* are reported to be related to retinal disorders [24, 25]. *ZBTB16* encodes a protein that is highly expressed in the brain, and polymorphisms in this gene are used as a marker for attention deficit hyperactivity disorder, a neuropsychiatric condition [26]. It is intriguing to note that the enriched signaling in neuronal and retinal genes are identified through epistasis evaluation between SNPs and CpG sites, but not through separate analysis of the main effect in those data sets. This shines light on the importance of integrated analysis of omics data: considering multiple facets or measurement of a common object may improve the chance of catching the underlining signal. Further studies on these threads are necessary to discover the underlying biological mechanism.
